# From follicle to blastocyst: microRNA-34c from follicular fluid-derived extracellular vesicles modulates blastocyst quality

**DOI:** 10.1186/s40104-024-01059-8

**Published:** 2024-08-04

**Authors:** Camilla Benedetti, Krishna Chaitanya Pavani, Yannick Gansemans, Nima Azari-Dolatabad, Osvaldo Bogado Pascottini, Luc Peelman, Rani Six, Yuan Fan, Xuefeng Guan, Koen Deserranno, Andrea Fernández-Montoro, Joachim Hamacher, Filip Van Nieuwerburgh, Trudee Fair, An Hendrix, Katrien Smits, Ann Van Soom

**Affiliations:** 1 Department of Internal Medicine, Reproduction and Population Medicine, Faculty of Veterinary Medicine, Ghent University, 9820 Merelbeke, Belgium; 2 Department for Reproductive Medicine, Ghent University Hospital, 9000 Ghent, Belgium; 3 Laboratory of Pharmaceutical Biotechnology, Faculty of Pharmaceutical Sciences, Ghent University, B-9000 Ghent, Belgium; 4 Department of Animal Science, University of Tennessee, Knoxville, TN USA; 5 Department of Nutrition, Genetics and Ethology, Faculty of Veterinary Medicine, Ghent University, B-9000 Ghent, Belgium; 6 Institute of Crop Science and Resource Conservation, Plant Pathology, Rheinische Friedrich-Wilhelms-University of Bonn, 53115 Bonn, Germany; 7 School of Agriculture and Food Science, University College Dublin, Belfield, Dublin, Ireland; 8 Laboratory of Experimental Cancer Research, Department of Human Structure and Repair, Ghent University, B-9000 Ghent, Belgium

**Keywords:** Developmental competence, Extracellular vesicles, Maturation, MicroRNA, miR-34c, Oocyte

## Abstract

**Background:**

Within the follicular fluid, extracellular vesicles (EVs) guide oocyte growth through their cargo microRNAs (miRNAs). Here, we investigated the role of EVs and their cargo miRNAs by linking the miRNAs found in EVs, derived from the fluid of an individual follicle, to the ability of its oocyte to become a blastocyst (competent) or not (non-competent).

**Methods:**

Bovine antral follicles were dissected, categorized as small (2–4 mm) or large (5–8 mm) and the corresponding oocytes were subjected to individual maturation, fertilization and embryo culture to the blastocyst stage. Follicular fluid was pooled in 4 groups (4 replicates) based on follicle size and competence of the corresponding oocyte to produce a blastocyst. Follicular fluid-derived EVs were isolated, characterized, and subjected to miRNA-sequencing (Illumina Miseq) to assess differential expression (DE) in the 4 groups. Functional validation of the effect of miR-34c on embryo development was performed by supplementation of mimics and inhibitors during in vitro maturation (IVM).

**Results:**

We identified 16 DE miRNAs linked to oocyte competence when follicular size was not considered. Within the large and small follicles, 46 DE miRNAs were driving blastocyst formation in each group. Comparison of EVs from competent small and large follicles revealed 90 DE miRNAs. Cell regulation, cell differentiation, cell cycle, and metabolic process regulation were the most enriched pathways targeted by the DE miRNAs from competent oocytes. We identified bta-miR-34c as the most abundant in follicular fluid containing competent oocytes. Supplementation of miR-34c mimic and inhibitor during IVM did not affect embryo development. However, blastocyst quality, as evidenced by higher cell numbers, was significantly improved following oocyte IVM in the presence of miR-34c mimics, while miR-34c inhibitors resulted in the opposite effect.

**Conclusion:**

This study demonstrates the regulatory effect of miRNAs from follicular fluid-derived EVs on oocyte competence acquisition, providing a further basis for understanding the significance of miRNAs in oocyte maturation and embryonic development. Up-regulation of miR-34c in EVs from follicular fluid containing competent oocytes and the positive impact of miR-34c mimics added during IVM on the resulting blastocysts indicate its pivotal role in oocyte competence.

**Supplementary Information:**

The online version contains supplementary material available at 10.1186/s40104-024-01059-8.

## Background

During the growth of the ovarian follicle, the mammalian oocyte undergoes a series of changes, preparing for final oocyte maturation [1, 2]. At the antral follicle stage, the oocyte becomes surrounded by specialized somatic cells, and follicles are characterized by a cavity filled with fluid rich in regulatory molecules. As such, follicular fluid represents a crucial environment for oocyte growth [3–5]. The follicular fluid contains an array of biomolecules (hormones, growth factors, and metabolites) and extracellular vesicles (EVs) [6, 7] that support oocyte maturation by participating in the bidirectional oocyte-somatic cell communication during follicle development [3]. This communication is crucial to determine oocyte competence, which can be defined as the ability of an oocyte to be fertilized and develop into a blastocyst [8]. As such, the composition of the follicular fluid can reflect the oocyte's quality and developmental competence [9]. More specifically, the follicular EVs and their miRNA cargo are important in this respect, since they may serve as biomarkers for the oocyte’s integrity [10] or even for its competence to develop to a blastocyst.

Extracellular vesicles are particles released from cells, delimited by a lipid bilayer, that cannot replicate on their own and carry specific cargoes to target cells. As such, these particles mediate paracrine effects in many physiological and pathological mechanisms [11]. Studies in humans, cattle, and horses consistently demonstrated that follicular fluid-derived EVs participate in oocyte development [12–14]. Supplementation of EVs from follicular fluid during in vitro oocyte maturation (IVM) resulted in the improvement of bovine cumulus expansion [10, 15]. We recently demonstrated that supplementing IVM medium with EVs from preovulatory follicles improved the blastocyst rate [16]. These findings suggest that the EVs or the cargo molecules within follicular fluid-derived EVs potentially impact the oocyte's fate.

In both human and bovine, small antral follicles contain oocytes that are not yet fully grown (typically smaller than 110 µm in diameter as compared to fully grown oocytes of 120 µm), which generally display reduced developmental potential [17, 18]. As a result, there is a preference in assisted reproductive techniques (ART), such as in vitro fertilization, to aspirate only fully grown oocytes from larger antral follicles (5–8 mm in human and bovine) [19, 20]. Hence, during follicle growth, the growing oocyte synthesizes and stores proteins and transcripts essential for the early stages of embryo development [21, 22]. As the oocyte grows, the concentration of maternal miRNA dilutes, resulting in a loss of regulatory effect on the maternal transcript [23]. However, this regulatory activity is linked to miRNA stability, as some miRNAs are abundantly expressed during oocyte growth and can efficiently suppress their targets, participating after fertilization in the oocyte-to-embryo transition [23]. In fully grown oocytes, only minimal transcriptional activity is present, and gene expression regulation occurs primarily at the post-transcriptional level until transcription resumption occurs at the time of embryonic genome activation [24, 25]. Recent research has identified miRNAs contained in follicular EVs as molecules involved in the post-transcriptional regulation of genes related to follicle and oocyte development [12, 26]. However, the global miRNA signature during antral follicle development and the critical miRNA predictors of oocyte competence in follicular fluid remain largely unknown.

Here, large (5–8 mm) or small (2–4 mm) antral follicles were isolated by dissection, their follicular fluid was collected separately, and then their oocytes were individually matured, fertilized, and cultured individually until the blastocyst stage, in order to be able to directly link the initial follicular content with the ability of each individual oocyte to become a blastocyst or not. Our aim was to analyze the miRNA content from the follicular fluid-derived EVs with regard to follicular size and to identify the miRNA repertoire needed for oocyte developmental competence using next-generation sequencing. We identified bta-miR-34c as specifically present in EVs from the competent follicular fluid. Notably, a functional assay using mimics and inhibitors of miRNA-34c was used to demonstrate an effect on embryo quality, especially on total cell number and inner cell mass ratio. Our findings establish a direct correlation between follicular EV-derived miRNAs and oocyte competence, thereby enhancing our fundamental understanding of early embryonic development.

## Methods

### Media and reagents

Tissue culture media (TCM)-199-medium, gentamycin, and kanamycin were obtained from Life Technologies (Ghent, Belgium). Phosphate-buffered saline (PBS) was obtained from Gibco™ (Thermo Fisher Scientific, Waltham, MA, USA). Other chemicals were purchased from Sigma-Aldrich (Overijse, Belgium). All media were filtered before use (0.22 μm; GE Healthcare-Whatman, Diegem, Belgium).

### Follicular fluid and cumulus oocyte complexes (COCs) collection

**Fig. 1:**
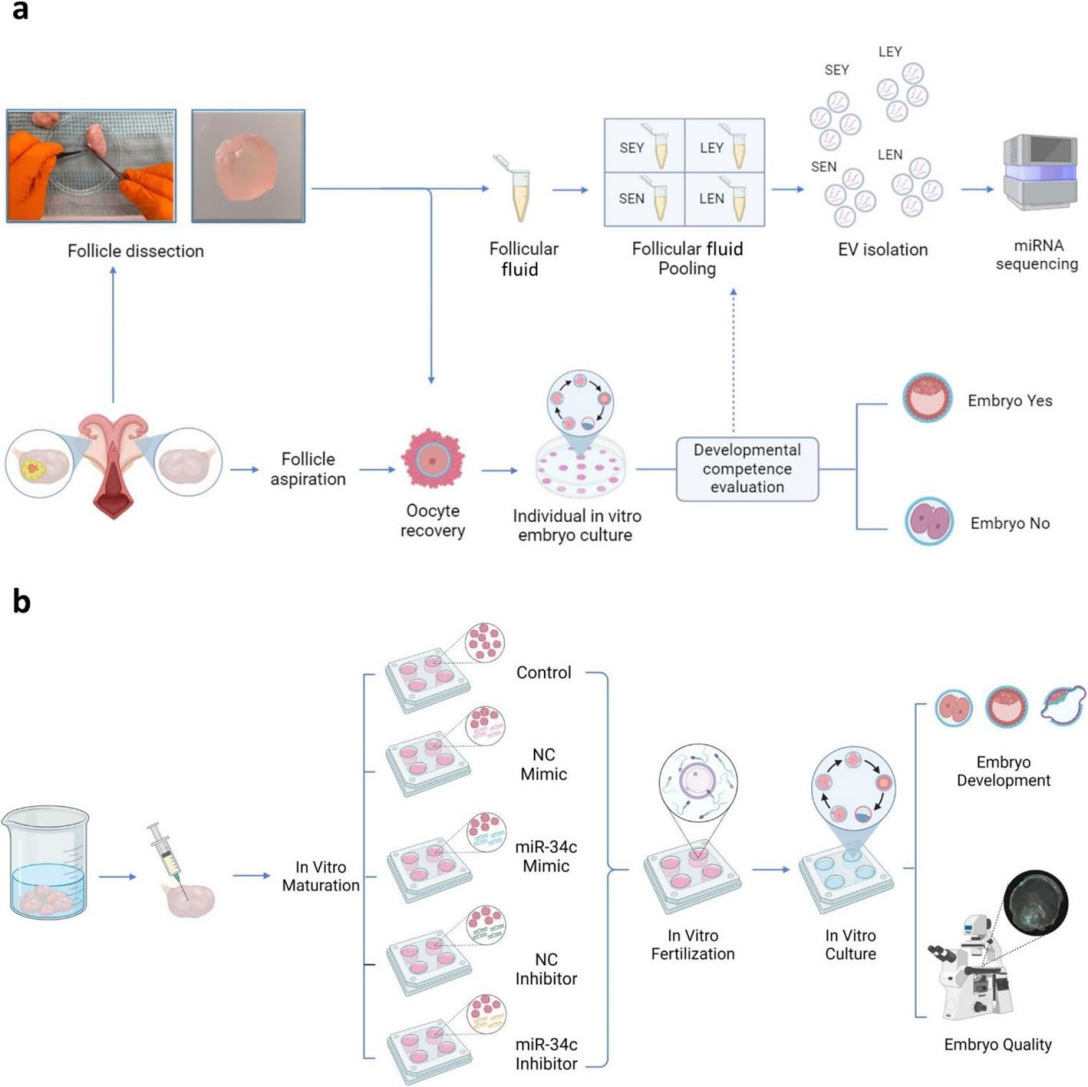
Visual representation of the experimental flow. **a** First experiment on the identification of critical miRNAs in oocyte developmental competence through the comparison: Small Embryo Yes (SEY); Small Embryo No (SEN); Large Embryo Yes (LEY); Large Embryo No (LEN). **b** Functional testing experiment on miR-34c. NC (negative control). Created with BioRender.com

The experimental design is summarized in Fig. 1. Bovine genital tracts were collected from a local slaughterhouse, and ovaries derived from non-pregnant uteri without signs of gross pathology were selected and processed within 2 h after collection. Briefly, the ovaries were disinfected with 70% alcohol and washed three times in warm (37 °C) physiological saline supplemented with kanamycin (25 mg/mL). Individual follicles, referred to as follicles dissected (FD), were isolated by dissection from selected ovaries without follicles ≥ 15 mm. For the FD group, the dissection of individual follicles was performed using a fine scissor. The dissected follicles were measured and categorized as small (S; 2–4 mm) and large (L; 5–8 mm). Cumulus-oocyte complexes were liberated by rupturing the follicles using a surgical blade under a stereomicroscope. Only COCs with ≥ 5 cumulus layers, homogeneous ooplasm, and compact cumulus investment (COC-A) or with minor granulation of the ooplasm and less compact cumulus cells (COC-B) were selected for in vitro embryo production. Once the COCs had been recovered, the follicular fluid of the FD was transferred individually into an RNase-free 2-mL tube and immediately placed on ice until all follicles were processed on a given day. Follicular fluid samples were centrifuged at 2,000 × *g* for 10 min at 4 °C to remove any cells and the supernatant was stored at –80 °C until EV isolation. For the follicle aspiration (FA) group, the same ovaries used for the FD group were used to collect COCs from the remaining follicles by aspirating antral follicles (between 2 and 8 mm) using an 18-gauge needle attached to a 10-mL syringe. The follicular fluid aspirated together with the COCs were transferred into a 2-mL tube. Once the COCs formed a pellet, the follicular fluid was discarded, and the COCs were recovered. As for the FD group, only COC-A and COC-B were selected for the in vitro embryo production for the individual control system (FA group).

### In vitro embryo production

For the individual culture systems (FA and FD), each droplet of medium contained a single COC or presumptive zygote to allow for the follow-up of the development of immature oocytes to the blastocyst stage. Our routine individual in vitro embryo production protocol was used as described previously [27]. Briefly, individual COCs were matured in 20 µL of IVM medium, made of modified bicarbonate-buffered TCM-199 with 50 µg/mL gentamycin and 20 ng/mL epidermal growth factor, covered with 8 mL of paraffin oil (SAGE, Cooper Surgical, Trumbull, CT, USA). In vitro maturation was carried out in Petri dishes (60 mm × 15 mm; Thermo Fisher Scientific, Waltham, MA, USA) for 22 h at 38.5 °C in 5% CO_2_ in humidified air. Before fertilization, photographs of all 222 COCs (173 COC-A and 49 COC-B) were captured upon collection (at 0 h) and after maturation (at 22 h), and cumulus expansion measurements and calculations were performed as previously described [28]. For in vitro fertilization, frozen-thawed spermatozoa from a proven fertile bull were separated using a Percoll gradient (45% and 90%; GE Healthcare Biosciences, Uppsala, Sweden) and washed with fertilization medium. The final concentration of 1 × 10^6^ sperm/mL was adjusted using in vitro Fertilization-Tyrode’s Albumin Lactate Pyruvate (IVF-TALP) medium containing bicarbonate buffered Tyrode’s solution, BSA (Bovine Serum Albumin, Sigma-Aldrich A8806; 6 mg/mL) and heparin (20 μg/mL). Matured oocytes were individually incubated in 20 µL IVF-TALP with spermatozoa and covered with 8 mL of paraffin oil for 21 h at 38.5 °C in 5% CO_2_ in humidified air. After in vitro fertilization, individual COCs were denuded from cumulus cells with a STRIPPER pipet holder and a 135-μm capillary (Origio, Cooper Surgical, CT, US). The presumptive zygotes were transferred into 20 µL droplets of synthetic oviductal fluid (SOF). enriched with non-essential and essential amino acids and ITS (5 μg/mL insulin; 5 μg/mL transferrin; 5 ng/mL selenium). SOF medium contained 0.1 mol/L NaCl, 7.1 mmol/L KCl, 1.3 mmol/L KH_2_PO_4_, 0.7 mmol/L MgSO_4_·7H_2_O, 0.6% (v/v) sodium lactate, 25 mmol/L NaHCO_3_, 0.02 mmol/L phenol red, 0.73 mmol/L sodium pyruvate, 1.78 mmol/L CaCl_2_·2H_2_O, 0.34 mmol/L trisodium citrate, 2.7 mmol/L myoinositol, 0.4 mmol/L glutamine, 0.4% BSA and 50 µg/mL gentamycin. The droplets were covered with 8 mL of paraffin oil and incubated at 38.5 °C in 5% CO_2_, 5% O_2_, and 90% N_2_ for 8 d. The individual droplets from the 2 groups (FA and FD) were always kept in the same Petri dish and not mixed in the same dish at any step of the in vitro embryo production system. Developmental outcome after individual embryo culture was evaluated at 2, 7, and 8 d post insemination (dpi).

### Follicular fluid classification and EVs isolation

Follicular fluid from individual follicles was pooled according to follicle size (small or large) and to embryo development outcome (embryo yes or embryo no) of the corresponding COC to reach a maximum volume of 200 µL (approximately from 15 follicles). Thus, 4 categories of follicular fluid were used for further molecular analysis, and 4 replicates were performed for each category of follicular fluid. Extracellular vesicles isolated from follicular fluid were obtained via single-step size exclusion chromatography (qEVsingle 70 nm smart columns, IZON™) as previously described [16]. The follicular fluid was loaded onto the column, followed by elution with 5 mL of freshly filtered PBS (pH: 7.4, 0.22-µm filtered). Extracellular vesicles-rich fractions 5 to 15 were pooled and loaded in Amicon^®^ Ultra-2 10-k centrifugal filters (UFC201024, Merck Millipore, Darmstadt, Germany) and centrifuged at 4 °C for 45 min at 3,000 × *g* using a swinging bucket rotor. The sample concentration was achieved by upside-down centrifugation at 4 °C for 2 min at 1,000 × *g*. EVs were retrieved from the flowthrough reservoir and stored at –80 °C until further analysis.

### Characterization of extracellular vesicles

**Fig. 2:**
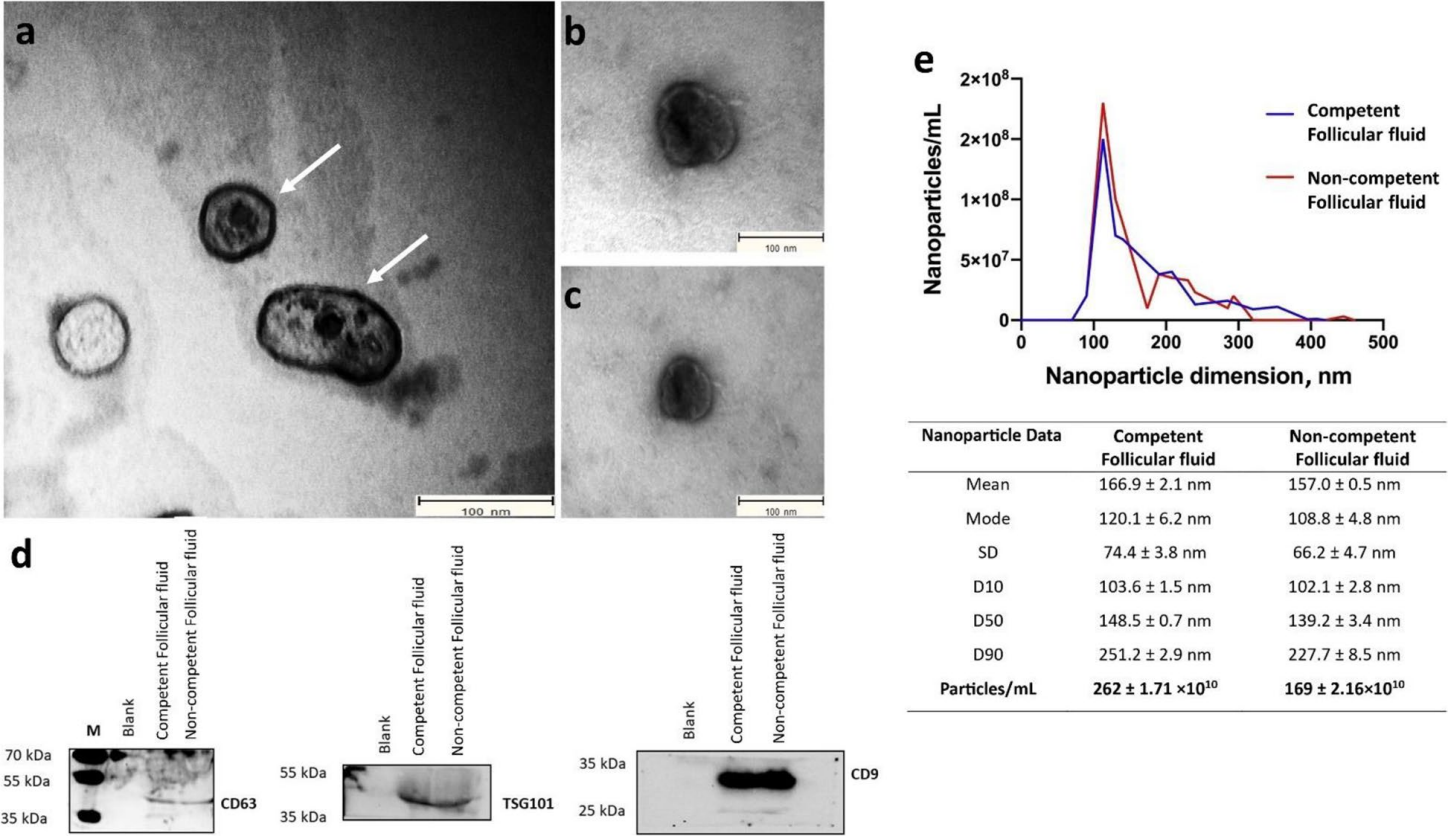
Identification and characterization of EVs isolated from the Competent and Non-competent Follicular fluid-EVs. Transmission electron micrograph image of the EVs (**a**) isolated from Competent Follicular fluid-EVs (**b**) and Non-competent Follicular fluid-EVs (**c**).  **d** Western blotting analysis of the EVs shows that Competent Follicular fluid and Non-competent Follicular fluid expressed traditional EV-associated markers CD63 (42 kDa), TSG101 (49 kDa) and CD9 (25 kDa). **e** Analysis of the particle size (nm) distribution of EVs isolated from Competent Follicular fluid and Non-competent Follicular Fluid EVs was determined by NTA. Measurements were taken in triplicates and reported in averaged Finite Track Length Adjustment (FTLA) concentration/size for the experiment. The mode, mean value, and SD of size and concentration were provided for each EV isolation. The value D50 represents the median size. Similarly, 90% of the distribution lies below the D90 value, and 10% of the population lies below the D10 value

The EV characterization methods used were described previously [29] and summarized in Fig. 2. Briefly, EVs were imaged using transmission electron microscopy (JEM 1400 plus and Zeiss EM 109 electron microscope) after being applied to copper mesh grids coated with a formvar/carbon film and stained with uranyl acetate. Extracellular vesicle particle concentration was measured with NanoSight LM10 using a 455 nm laser. In detail, three 60-s videos were recorded per sample, with settings adjusted for optimal measurements (dilution with PBS). Finally, western blotting was used to detect EV-specific proteins. The EV samples were suspended in a buffer and heated to denature the proteins. Subsequently, denatured proteins were separated by size using gel electrophoresis and transferred onto a nitrocellulose membrane (Bio-Rad, Hercules, California, USA). After blocking, the membrane was incubated overnight to specific primary antibodies targeting proteins of interest, including CD63 (ab68418, Abcam, Cambridge, UK), CD9 (D3H4P, Cell Signaling Technology, Boston, MA, USA), TSG101 (sc-7964, Santa Cruz, Oregon, CA, USA), Argonaute 2 (AGO-2; ab32381, Abcam, Cambridge, UK), and Apolipoprotein A1 (ApoA-I; sc-376818). Following the primary antibody incubation, unbound antibodies were removed by washing, and secondary antibodies were applied. Anti-mouse IgG 9 (GE Healthcare, Buckinghamshire, UK) and anti-rabbit IgG (GE Healthcare, Buckinghamshire, UK) secondary antibodies were used accordingly, based on the primary antibody types. Following washing to remove unbound secondary antibodies, a chemiluminescence substrate (WesternBright Sirius, Advansta, Menlo Park, CA, USA) was added to the membrane, and imaging was performed using Proxima 2850 Imager (IsoGen Life Sciences, De Meern, The Netherlands).

### Isolation of total RNA, including miRNAs

For all four follicular fluid categories, EV’s total RNA was isolated using Plasma/Serum and Exosomal RNA Purification Kit (Slurry Format; Norgen Biotek Corp., Ontario, Canada) according to the Manufacturer’s instructions. Total RNA was eluted in 20 µL of elution solution. Before further processing, the concentration and integrity of the total RNA were assessed using an RNA 6000 Pico Chip (Agilent Technologies) and a Quant-iT RiboGreen RNA Assay kit (Life Technologies), respectively.

### Small RNA library preparation and deep sequencing

Small RNA library construction and deep sequencing were conducted using the Tailormix v2 kit from SeqMatic. These libraries were then combined into pools and subjected to sequencing at the Illumina Miseq. Afterward, rigorous quality control measures including assessing sequence quality, sequencing depth, clonal read duplication rates, alignment quality, and potential bias in nucleotide composition were performed.

### Small RNA-seq data analysis

To identify known and novel miRNAs, the miRPro pipeline (v1.1.4) [30] with the latest miRBase (v22.1) [31] data and the bovine genome (*Bos taurus* ARS-UCD1.2, ENSEMBL release 105) were used. A total of five comparisons were performed to evaluate differential expression (DE) of EV–derived miRNAs related to follicle size (large vs. small) and oocyte competence (embryo yes vs. no): LEY (Large, Embryo YES) vs. LEN (Large, Embryo No), SEY (Small, Embryo Yes) vs. SEN (Small, Embryo No), LEY vs. SEY, LEN vs. SEN and LSEN (Large Small, Embryo No) vs. LSEY (Large Small, Embryo Yes). The identification of DE miRNAs was performed in R (v4.1.2) using the edgeR (v3.36) package. A miRNA was considered DE when the Benjamini–Hochberg adjusted *P* value was < 0.05 and the absolute value of the log_2_ foldchange was ≥ 1.

### Gene Ontology (GO) pathway enrichment analysis

Candidate target genes for the significantly DE miRNAs (known + novel) were predicted with miRanda (v3.3a) [32] using the miRNA and 3′-UTR sequences. The most relevant target candidates were filtered based on their score (> 155) and energy (< –20). The analysis was done for all DE comparisons. To further explore the biological role of these results, enrichment analyses were done in R (v4.1.2) using the fgsea (v1.20.0), EnrichmentBrowser (v2.24.2), and simplifyEnrichment (v1.4.0) packages [33]. Pathways with a *P* value ≤ 0.05 were considered statistically significant.

### miRNA functional analysis

#### Labeling and uptake

A group of 60 COCs was cultured in maturation medium supplemented with 1 μmol/L of fluorescently labeled negative control (NC) miRNA mimics (specifically, miRCURY LNA miRNA Mimic—5′ FAM, Product No. 339173, Qiagen). A control group was included by adding an equal volume of RNase-free water to the maturation medium. After maturation, COCs were subjected to 2 washes in PBS–BSA and fixed at room temperature for 20 min in 4% paraformaldehyde. Subsequently, all COCs underwent staining with Hoechst 33342 (diluted at a 1:100 ratio in PBS) for 10 min. An additional group of 60 COCs, along with a control group, were cultured as previously described. Following maturation, cumulus cells were removed by gentle pipetting, and the resulting denuded oocytes were fixed and stained with Hoechst 33342, as described above. The COCs and denuded oocytes were imaged using a ZEISS Confocal Microscope (ZEISS LSM 980, Zeiss Microscopy). This experimental procedure was repeated three times, and each replicate involved the assessment of 5–10 COCs.

#### RT-qPCR analysis

Double strands mimic of miR-34c (Sangon Biotech) was supplemented to the IVM medium with a final concentration of 1 µmol/L. A control group was an equal volume of RNase-free water to the IVM medium was included. After maturation, total RNA (including small RNA) was isolated from cumulus cells and denuded oocytes with and without miR-34c mimic supplementation (3 replicates, 20 oocytes/group) using miRNeasy Micro Kit (Qiagen). The RNA was subsequently eluted in 14 μL of RNase-free water and quantified using a Nanodrop™ (ND-100 spectrophotometer). For cDNA preparation, a Reverse Transcription miRCURY LNA miRNA PCR Starter Kit from Qiagen (Germantown, USA) was used. RT-qPCR experiments were carried out on a BioRad CFX 96 PCR detection system. In detail, 2.5 μL of template cDNA were combined with 10 μL of SYBR^®^ LNA miRNA PCR Assays (Qiagen, Germantown, USA), along with 0.3 mmol/L of each primer, in a final volume of 20 μL. The PCR program consisted of an initial denaturation step at 95 °C for 3 min, followed by 40 cycles of denaturation at 95 °C for 5 s, and then a combined primer annealing-extension step at specific annealing temperatures for 30 s. Subsequently, a melting curve analysis was conducted by gradually increasing the temperature from 70 to 95 °C in 0.5 °C increments for 5 s, ensuring the presence of a single specific peak for each primer pair. The miR-92a was used to normalize miR-34c expression [34, 35]. All RT-qPCR reactions were performed in triplicate, and data analysis was conducted using the 2^−^^ΔΔCt^ method. The list of the primers used for the RT-qPCR is listed in Table 1. The data were analyzed using GraphPad Prism Software (GraphPad 103 Software Inc., San Diego, CA, USA).

**Table 1:** Sequence of the primers used for RT-qPCR

Target miRNA	Accession	Sequence (5′→3′)	Qiagen GG Cat. No.
bta-miR-34c	MIMAT0003854	AGGCAGUGUAGUUAGCUGAUUG	YP02103323
bta-miR-92a-3p	MIMAT0000092	UAUUGCACUUGUCCCGGCCUGU	YP00204258

#### Supplementation of mimics and inhibitors

Mimics and inhibitors of miR-34c (Sangon Biotech) were supplemented to the IVM medium as described above. In parallel, NC of mimic (sequence 5′→3′: UUGUACUACACAAAAGUACUG) and inhibitor (sequence 5′→3′: CAGUACUUUUGUGUAGUACAA) were supplemented to the IVM medium. The final experimental design included a control group supplemented with an equal volume of RNA-free water. For this experiment, oocytes collected from small antral follicles (2–4 mm) were used. Only COC-A and COC-B were selected for in vitro embryo production (four replicates). Briefly, COCs were randomly allocated to control (*n* = 353), miR-34c mimic (*n* = 404), miR-34c inhibitor (*n* = 393), NC mimic (*n* = 346), and NC inhibitor (*n* = 369). Maturation media were prepared as mentioned above, and IVM was performed by culturing approximately 60 COCs in 500 µL of maturation medium for 22 h at 38.5 °C in 5% CO2 in four-well plates (Nunc™, Thermo Fisher Scientific, Waltham, MA, USA). Fertilization and embryo culture protocols were performed as previously described, except that in vitro fertilization occurred in 500 µL of fertilization medium, and zygotes were cultured in groups of 25 in 50 µL of SOF covered with paraffin oil. Developmental outcome after individual embryo culture was evaluated at 2, 7, and 8 dpi. Subsequently, all blastocysts (8 dpi) were collected for embryo quality assessment through differential apoptotic staining.

### Embryo quality assessment using differential staining

Differential apoptotic staining was performed as described by Wydooghe et al. [36]. Briefly, 260 blastocysts at 8 dpi were fixed in a 2% paraformaldehyde solution at room temperature for 20 min. Subsequently, they were stored in phosphate-buffered saline containing 0.5% BSA at 4 °C. The first immunofluorescent staining was performed using CDX2, a transcription factor exclusive to trophectoderm (TE) cells. This was followed by active Caspase-3 staining to detect apoptosis, and these procedures were further complemented with 0.1% Hoechst 33342 staining. Quantitative assessments included the evaluation of the following parameters: the number of TE cells, the number of inner cell mass cells (ICM), the total cell number (TCN), the ratio of ICM to TCN, and the overall count of apoptotic cells (AC), including their ratio (calculated as AC divided by TCN). These assessments were conducted using fluorescence microscopy (Leica DM 5500 B) equipped with a triple bandpass filter and performed by a single observer.

### Statistical analyses

Data have been manually collected and exported to Microsoft Excel (Microsoft Corp.), where the PivotTables function (Microsoft Excel) was used for data exploration and organization. The statistical analyses were performed using R-core (version 4.2.1; R Core Team). The effects of collection method (FD, FA) and follicle size on developmental parameters (embryo development at 2 and 8 dpi) were fitted in generalized mixed‐effects models in which the cow and replicate were set as random and expressed as a percentage from presumed zygotes. The differences between groups were assessed using Tukey’s post hoc test. Results are expressed as least-squares means and SEM and the significance level was set at *P* < 0.05.

## Results

### Follicular dissection has no adverse effect on embryo development

The developmental competence of COCs retrieved by follicular dissection (FD) was compared with that of COCs collected by routine follicular aspiration (FA). Interestingly, COCs collected after FD showed a higher proportion of quality A COCs (78.0% ± 3.3%) than FA COCs (51.6% ± 3.6%, Additional file 1, Table S1). The area of FD COCs was significantly greater than in the FA group, both before (FD = 75,529 µm^2^ and FA = 17,493 µm^2^; *P* = 0.033) and after maturation (FD = 119,426 µm^2^ and FA = 28,993 µm^2^; *P* = 0.031; Additional file 1, Table S1). However, the relative cumulus expansion among the two groups was not significantly different (*P* = 0.2). When comparing developmental competence in an individual culture system, we observed no differences in terms of cleavage rates and blastocyst yield in FD vs. FA (Additional file 1, Table S1).

### Follicular fluid-derived EVs contain miRNAs controlling oocyte fate

**Fig. 3:**
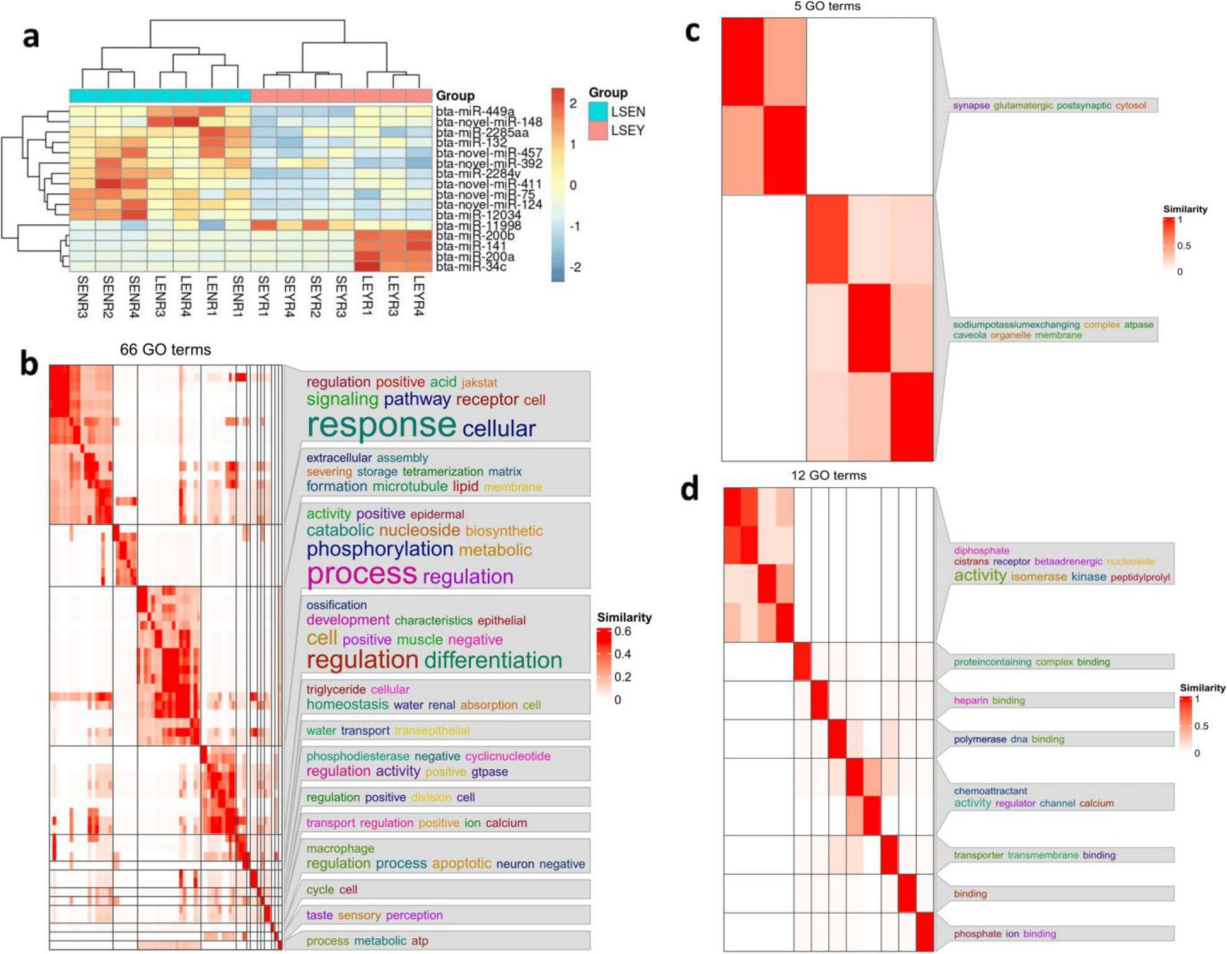
The miRNA profile of EVs derived from competent (LSEY) and non-competent follicular fluid (LSEN). **a** Heatmap generated by clustering of the DE miRNAs in EVs derived from competent and non-competent follicular fluid. Red, up-regulation; blue, down-regulation compared to the mean expression over all samples. **b–d** GO enrichment of DE miRNAs present in EVs isolated from either competent and non-competent follicular fluid (LSEY vs. LSEN). Semantic similarity scores were determined for significant Gene Ontology (GO) terms and their corresponding categories (biological process [BP], cellular component [CC], and molecular function [MF]) using Schlicker’s Relevance method. Subsequently, GO terms were grouped through binary cut enrichment and organized into clusters based on their associations with (b) BP, (c) CC, and (d) MF

**Fig. 4:**
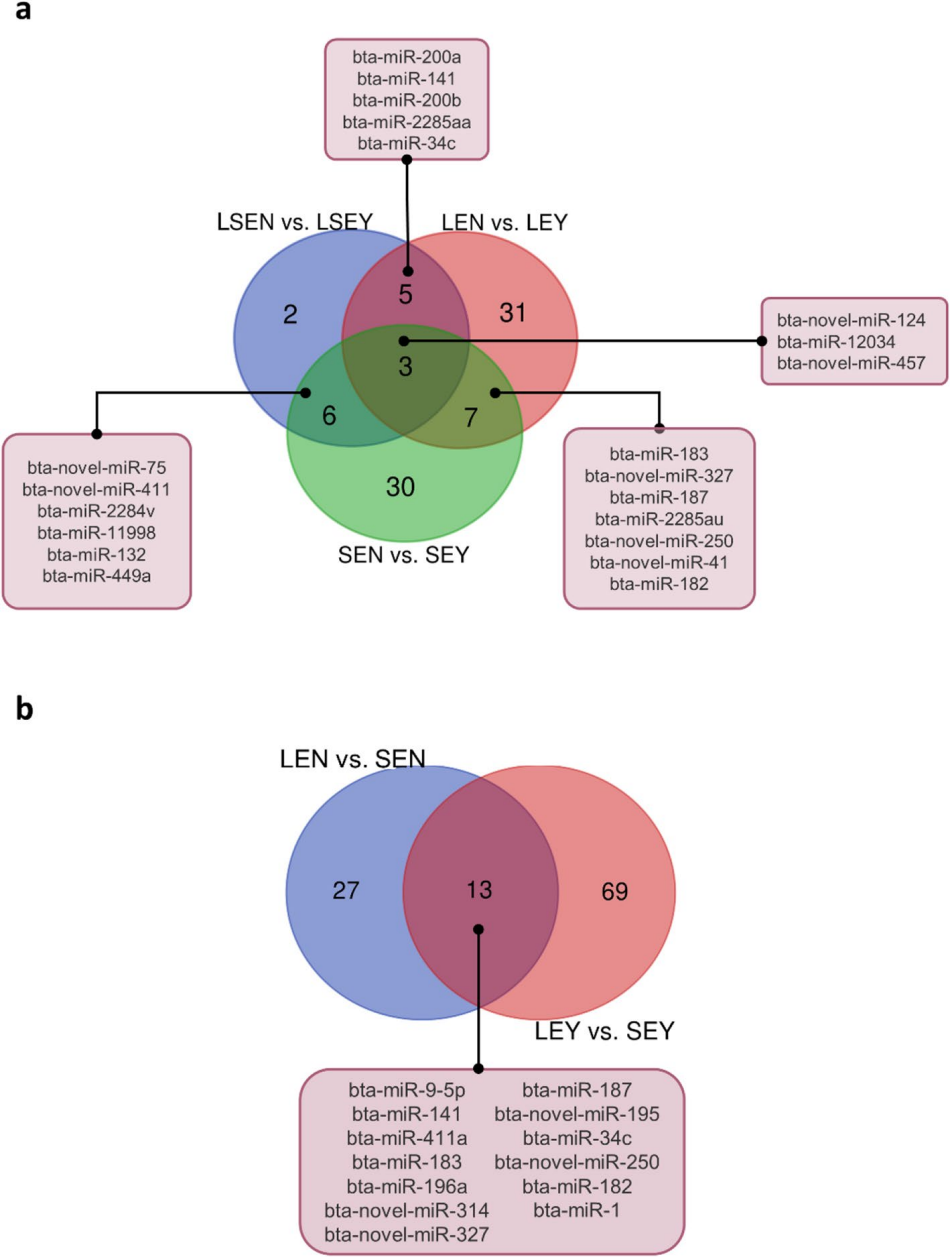
Venn diagram showing the common differentially expressed miRNAs in EVs isolated from different follicular fluid groups. **a** Comparisons to characterize the miRNAs involved in oocyte developmental competence. Large Small Embryo No (LSEN) vs. Large Small Embryo Yes (LSEY) compared with Large Embryo No (LEN) vs. Large Embryo Yes (LEY) and Small Embryo No (SEN) vs. Small Embryo Yes (SEY). **b** Comparisons to identify the miRNAs involved in oocyte developmental competence during antral follicle growth

To investigate the miRNAs participating in oocyte developmental competence, we made pairwise comparisons between competent and non-competent oocytes (Embryo Yes (EY) vs. Embryo No (EN)), considering the size of the original follicle (Large (L; 5–8 mm) vs. Small (S; 2–4 mm)). Comparison of LEY vs. LEN resulted in 16 up- and 30 downregulated miRNAs, and SEY vs. SEN in 22 up- and 24 downregulated miRNAs (Table 2). Annotation of the novel and known DE miRNAs is given in Additional file 1 (Table S2 and S3). The GO analyses of target genes in LEY vs. LEN and SEY vs. SEN are summarized in Additional files 2 and 3 (Fig. S1 and S2). Further comparison between competent and non-competent oocytes without considering the size of origin (LSEY vs. LSEN) resulted in 16 DE miRNAs with 6 novel (all upregulated) and 10 known miRNAs (5 up- and 5 down-regulated, Additional file 1, Table S4). For the DE novel miRNAs, no match was found with previously known bovine seed regions and homologs in other species. Hierarchical clustering analysis confirmed the categorization of DE miRNAs into competent follicular fluid and non-competent follicular fluid (Fig. 3a). Target analysis by miRanda revealed 12,980 genes targeted by the 16 DE miRNAs, with GO gene sets enriched in cell regulation, cell differentiation, cell cycle, metabolic process regulation, and ATP binding (Fig. 3b–d). Across comparisons (LEY vs. LEN, SEY vs. SEN, and LSEY vs. LSEN), 3 shared DE miRNAs (bta-novel-miR-124, bta-miR-12034, and bta-novel-miR-457) were found (Fig. 4a). We additionally identified 5 DE miRNAs (bta-miR-200a, bta-miR-141, bta-miR-200b, bta-miR-2285aa, and bta-miR-34c) that are associated with acquisition of oocyte competence in large follicles (Fig. 4a). Of those, bta-miR-34c showed the highest expression in LSEY vs. LSEN (logFC = 3.09) (Additional file 1, Table S4). Analysis of miRbase revealed that bta-miR-200a/b and bta-miR-141 belong to the same family (miR-8). Conversely, we identified 6 DE miRNAs (bta-novel-miR-75, bta-novel-miR-411, bta-miR-2284v, bta-miR-11998, bta-miR-132 and bta-miR-449a) that are associated with the acquisition of oocyte competence in small follicles (Fig. 4a).

**Table 2:** Number of DE novel and known miRNAs among the four follicular fluid categories

Comparison	Nr. DE miRNAs	Upregulated miRNAs	Downregulated miRNAs	Novel miRNAs	DE miRNA–target genes
LEY vs. LEN	46	16	30	13	6,598
LEN vs. SEN	40	14	26	14	7,308
SEY vs. SEN	46	22	24	16	7,047
LEY vs. SEY	90	46	44	31	13,974

DE miRNAs were identified using miRBase (v22.1) and *Bos taurus* genome (ARS-UCD1.2, ENSEMBL release 105) as reference. Candidate target genes for the significantly DE miRNA (known + novel) were predicted with miRanda (v3.3a) using the miRNA and 3′-UTR sequences, with the following thresholds: minimum score = 155 and maximum energy = –20

### Follicle size affects oocyte development and the expression of miRNAs within follicular fluid-derived EVs

**Fig. 5:**
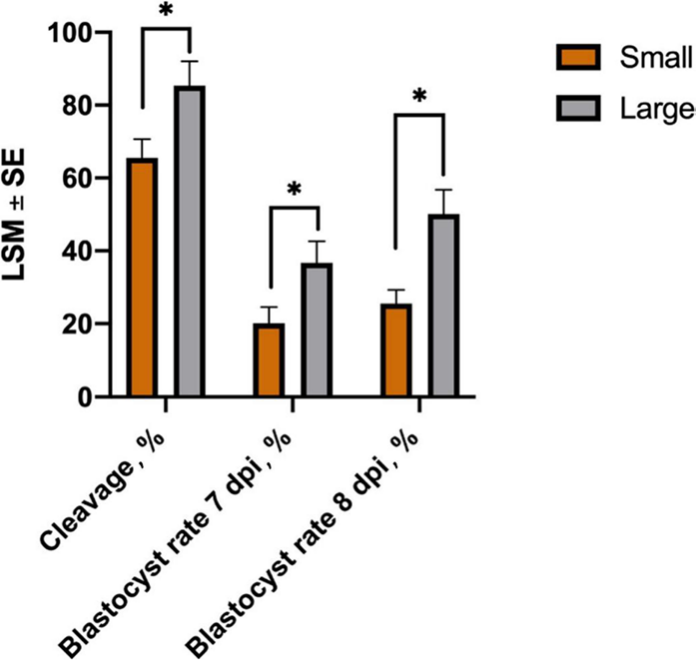
Effect of follicle size on oocyte developmental competence. Cleavage, 7 and 8 dpi blastocyst rates are expressed as a percentage of presumed zygotes. Dissected follicles were measured in size, classified as small (2–4 mm) or large (5–8 mm), and the respective oocytes were collected and used for in vitro embryo production using an individual maturation-fertilization-culture system. Results are expressed as least-square mean ± SE, *P < 0.05

Evaluation of the effect of follicle size (S or L) on embryo development using an individual culture showed that the cleavage rate was higher for oocytes originating from large follicles than for those from small ones (85.4% ± 6.6% and 65.5% ± 5.2%, respectively; *P* = 0.031; Fig. 5). Large follicles also resulted in a greater yield of blastocysts compared to small follicles, both at 7 dpi (36.7% ± 5.9% vs. 20.1% ± 4.4%, *P* = 0.01) and 8 dpi (50.1% ± 6.7% vs. 25.5% ± 3.8%, *P* = 0.01; Fig. 5). Comparing miRNA data from SEY vs. LEY, 90 DE miRNAs were identified (46 up- and 44 down-regulated; Table 2). Interestingly, 28% of the known miRNAs were located on Chr21 and clustered in the same region (Additional file 1, Table S5). MiRanda analysis revealed 13,974 genes targeted by DE known and novel miRNAs (Table 2), with GO gene sets enriched in transport regulation, signaling pathway response, and kinase activity (Additional file 4, Fig. S3). Differential expression analysis of the data identified bta-miR-34c, bta-miR-34b, bta-miR-200c, and bta-miR-141 as significantly downregulated in SEY (Additional file 1, Table S5).

Finally, we investigated the effect of follicle size on poor oocyte competence by comparing LEN vs. SEN. Among the DE novel miRNAs, we identified the homologs of two novel miRNAs (bta-novel-miR-215 and bta-novel-miR-39) in other species (ggo-mir-203b, rno-mir-203b, and oan-mir-145, respectively). The remaining 12 novel miRNAs have yet to be annotated (Additional file 1, Table S6). The miRanda tool predicted 7,308 genes targeted by DE known and novel miRNA (Table 2), resulting in GO gene sets enriched in metabolic processes, negative regulation of signaling pathways, homeostasis regulation, and membrane vesicle component (Additional file 5, Fig. S4). We found 13 shared DE miRNAs across the comparison LEY vs. SEY and LEN vs. SEN, including bta-miR-34, bta-miR-411a, and bta-miR-141, which were downregulated in SEN and upregulated in LEY (Fig. 4b).

Overall, bta-miR-34c was significantly more abundant in all follicular fluid groups associated with competent oocytes. Due to its positive association with oocyte competence and evidence from the literature supporting its role in embryonic development and cell cycle progression [37–45], we selected bta-miR-34c for further functional studies.

### Cumulus-oocyte complexes take up miR-34c mimic during oocyte IVM

**Fig. 6:**
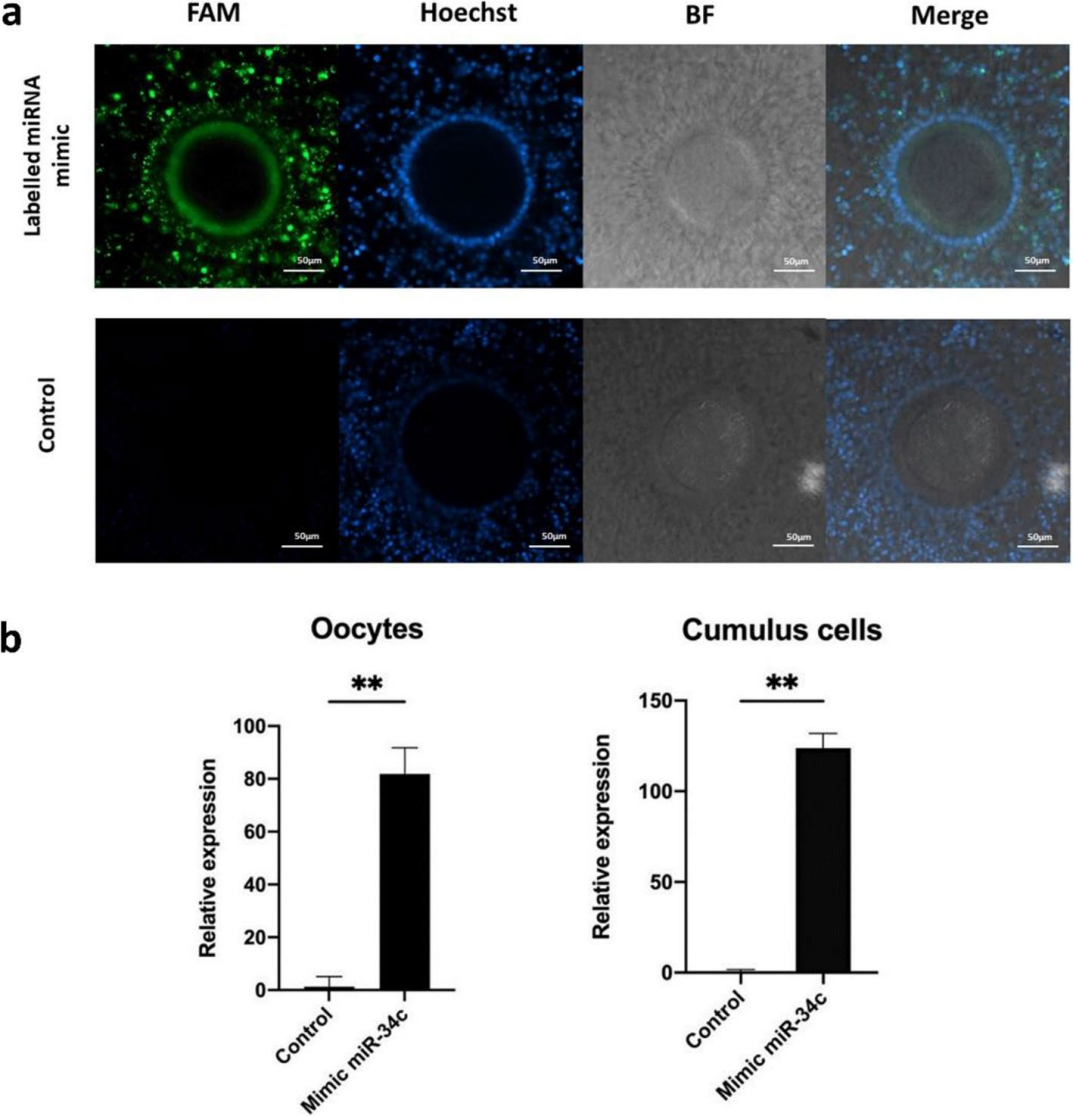
Validation of microRNA mimics uptake by cumulus-oocyte complexes (COCs) during in vitro oocyte maturation. **a** Bright-field and fluorescence images of COCs matured in vitro with or without labeled miRNA mimic supplementation. After 22 h of in vitro maturation, along with labeled mimics (5′ FAM, green) or PBS (Control), COCs were washed, fixed, and stained with Hoechst (blue) to visualize the nuclei. The merged image demonstrates the uptake of green fluorescent–labeled mimics by COC. A control with PBS incubated with bovine COCs showed no green fluorescence. **b** Mimics uptake was confirmed by quantification of miR-34c in cumulus cells and oocytes after in vitro maturation with or without miR-34c mimic supplementation. Quantification was calculated based on the raw Cq values. Data from three replicates of all the samples are shown, ^**^P < 0.01, ANOVA with Tukey’s HSD post-hoc test

Both fluorescence microscopy and RT-qPCR demonstrated the uptake of miRNA mimics by COCs during IVM. Following 22 h of incubation with fluorescently labeled double RNA strand mimics, evaluation under confocal microscopy revealed a high concentration of labeled mimics in the oocyte cytoplasm (Fig. 6a and Additional file 6, Fig. S5), indicating that mimics passed through the cumulus cells and the zona pellucida. RT-qPCR further confirmed significantly higher miR-34c expression in both cumulus cells (FC = 123.79) and denuded oocytes (FC = 81.86) after IVM in the presence of miR-34c mimics, compared to the non-supplemented control (*P* = 0.01 and *P* = 0.009, respectively, Fig. 6b).

### MicroRNA-34c mimics and inhibitors modulate embryo quality

**Fig. 7:**
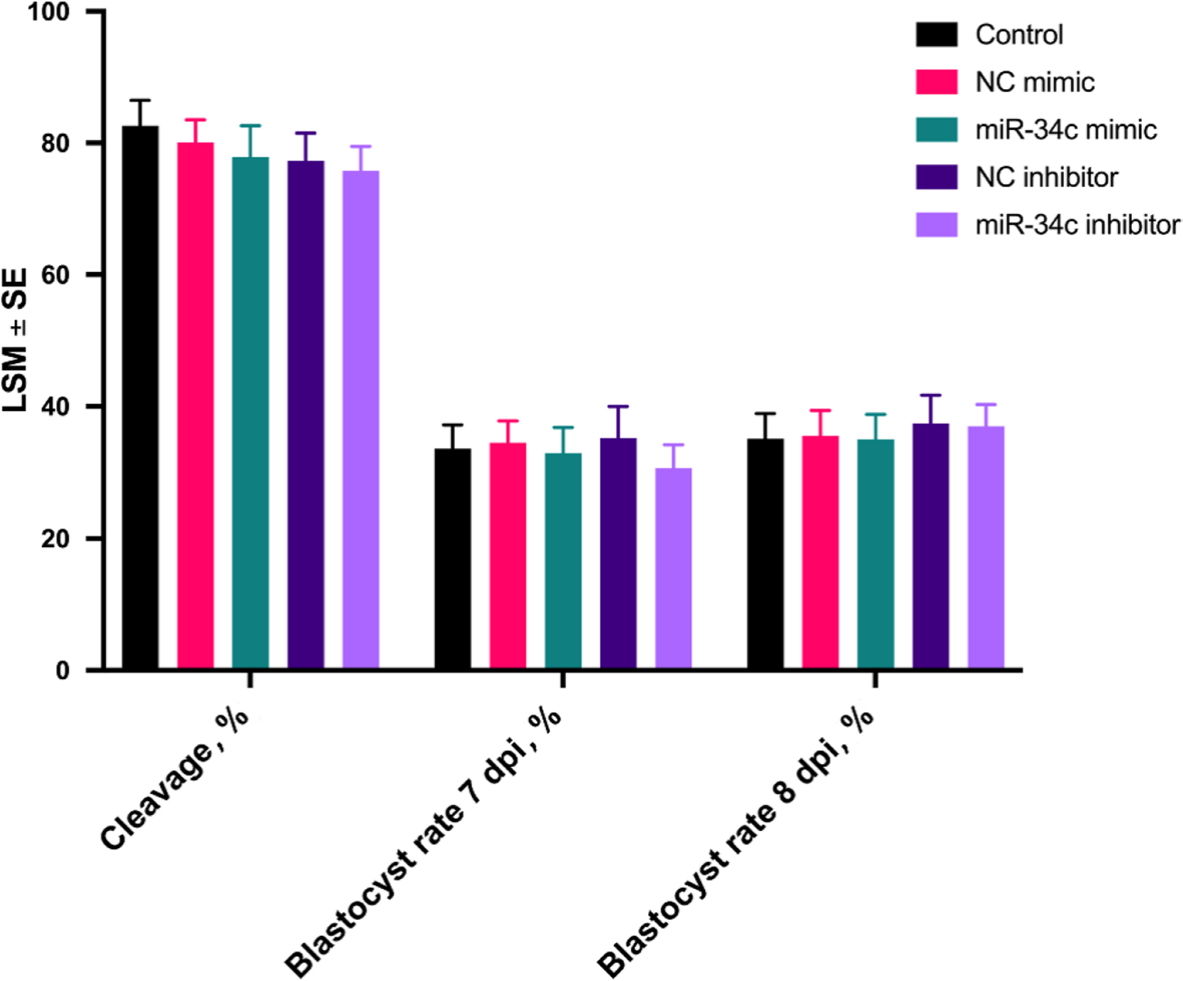
Blastocyst development of bovine cumulus-oocyte complexes treated with miR-34 mimic/inhibitor. Cleavage, 7 and 8 dpi blastocyst rates are expressed as a percentage of presumed zygotes. For the miRNA mimic experiment, maturation media were not supplemented (Control) or supplemented with control mimics (NC mimics) or miRNA-34c mimics. For the miRNA inhibitor experiment, culture media were not supplemented (Control) or supplemented with control inhibitors (NC inhibitor) or miRNA-34c inhibitor. Results are expressed as least-square mean ± SE

Based on the results from our preliminary study, we used COCs from small follicles (2–4 mm) to test the hypothesis that miR-34c can improve their developmental competence. Supplementation of miR-34c mimics and inhibitors during IVM did not affect cleavage and blastocyst rates at 7 and 8 dpi in comparison to the control, negative control (NC) mimics, and NC inhibitor (*P* > 0.05; Fig. 7). In contrast, differential apoptotic staining revealed a positive effect of miR-34c on blastocyst quality. Notably, miR-34c mimics supplementation during IVM resulted in blastocysts with a higher total cell number (TCN) (143.2 ± 5.3), inner cell mass (ICM) (90.3 ± 5.3), and ICM/TCN ratio (62.0 ± 2.5) than the control (132.9 ± 3.2, 75.6 ± 3.0 and 56.4 ± 1.5, respectively, *P* < 0.05, Table 3). In line with these findings, supplementation of IVM media with a miR-34c inhibitor resulted in a lower TCN (122.0 ± 4.3), ICM (64.7 ± 4.2), and ICM/TCN (49.7 ± 2.4) ratio than the control (*P* < 0.05).

**Table 3:** Effect of miR-34c mimic supplementation during vitro maturation on embryo quality

Treatment	No. of blastocysts	TCN	TE	AC	ICM	ICM/TCN ratio, %	AC/TCN ratio, %
Control	73	132.9 ± 3.2^a^	57.2 ± 2.2	6.3 ± 0.35	75.6 ± 3.0^a^	56.4 ± 1.6^a^	4.8 ± 0.2
NC mimic	44	135.0 ± 3.6^a^	60.2 ± 4.7	6.5 ± 3.2	75.2 ± 5.0^a^	54.9 ± 2.9^a^	5.3 ± 1.4
miR-34c mimic	46	143.2 ± 5.3^b^	53.0 ± 3.1	7.7 ± 1.1	90.3 ± 5.3^b^	62.0 ± 2.5^b^	5.6 ± 0.8
NC inhibitor	50	136.5 ± 3.8^a^	59.9 ± 5.1	7.0 ± 2.7	71.9 ± 3.2^a^	54.4 ± 1.8^a^	4.8 ± 2.2
miR-34c inhibitor	47	122.0 ± 4.3^c^	57.3 ± 3.4	6.8 ± 0.6	64.7 ± 4.0^c^	49.7 ± 0.5^c^	5.8 ± 2.4

*TCN* Total cell number, *TE* Trophectoderm cells, *ICM* Inner cell mass, *AC* Apoptotic cells, *ICM/TCN* ratio, and *AC/TCN* ratio of d 8 blastocysts

^a–c^Different superscripts per column represent statistical differences (*P* < 0.05). Results are expressed as least square means ± standard error (LSM ± SE)

## Discussion

In this study, we were able to identify 16 miRNAs that were differentially expressed in EVs of antral follicles, containing an oocyte that was able to develop into a blastocyst. Here, we argue that those miRNAs are candidate molecules to serve as a follicular biomarker for competent oocytes or to improve oocyte competence during IVM. We could confirm that bta-miR-34c when added during IVM, could improve the resulting blastocyst quality in terms of TCN and ICM. Central in our achievement was (a) our optimized EV isolation method [16, 29], which allowed for the successful extraction of EVs from limited volumes (9–45 µL) of fluid from individual antral follicles and efficient characterization of the EV-derived miRNAs and (b) the individual follicle to blastocyst follow-up, which allowed to relate the outcome of the oocyte back to its original follicle. Moreover, we demonstrated convincingly that miRNA mimics are taken up by cumulus cells and penetrate the zona pellucida of oocytes during IVM without transfection reagents. Here, in agreement with previous studies [46–48], we confirmed that antral follicle size is a crucial element for selecting competent oocytes, with oocytes originating from large antral follicles (5–8 mm) being superior to those from small follicles (2–4 mm); but more importantly, we demonstrated for the first time that follicular fluid-derived EVs contain miRNAs that are indicative for further oocyte competence. Recent studies have also highlighted the potential influence of miRNAs from follicular fluid-derived EVs on the oocyte. In pigs [49], specific miRNA profiles were identified in EVs from follicular fluid associated with oocyte integrity. In this research, lissamine green B, a vital stain that efficiently detects cellular membrane damage, was used to assess oocyte membrane integrity [50]; thus, ruling out severely compromised oocytes, but it cannot be used to assess oocyte competence since not all membrane intact oocytes have the ability to form a blastocyst. In our study, we focused on linking the miRNA profiles of EVs present in follicular fluid with the competence of the oocytes contained therein to ultimately develop into blastocysts. Although oocyte integrity is one of the factors influencing oocyte competence, our study did not identify any significant DE miRNAs shared in common with the research discussed above. A recent study in bovine [51] profiled the miRNAs of EVs from “good” and “poor” bovine follicular fluid. In line with our approach, the follicular fluid classification used in this study was based on the oocyte's ability to form a blastocyst, but the main difference was that follicular fluid was not collected from individual follicles but from individual ovaries, and the assignment of follicular fluid as being good or poor was merely based on the capability of the group of oocytes derived from a given ovary to produce blastocysts, with no further details on the cut-off value for blastocyst development. Since the follicular fluid samples were collected only on an individual ovary basis, in that study, the miRNA profile did not link oocyte developmental competence with the individual follicular fluid environment in which oocytes obtained this competence.

The use of an individual culture system allowed the follow-up of the oocyte from the follicle over IVM until the blastocyst stage [8, 27]. As such, we could link the miRNA profile of the follicular EVs to oocyte developmental competence (Fig. 1). The general comparison between competent and non-competent follicular fluid (LSEY vs. LSEN) revealed enrichment in miRNAs involved in cell regulation, cell differentiation, cell cycle, metabolic process regulation, and ATP binding (Fig. 3b–d). Among the 16 DE miRNAs, miR-34c was the most significantly upregulated in LSEY (logFC = 3.09). The observed miR-34c expression among the comparisons suggests a crucial role of miR-34c in determining oocyte developmental competence, coinciding with numerous studies that support its role in the cell cycle and embryo development [34–42]. The dysregulation of the miR-34 family in somatic cells, notably observed in cancers like Merkel cell carcinoma, suggests its role as a tumor suppressor through involvement in tumor-associated processes like epithelial-mesenchymal transition and the p53 network [37, 38, 52, 53]. Furthermore, a recent investigation on breast cancer demonstrated that miR-34c targets CCND1, CDK4, and CDK6, pivotal cell cycle regulators that prompt arrest at the G2/M stage [54]. The role of miR-34c in reproduction was examined in cattle, mice, and humans [39–41]. In mice and humans, miR-34c is absent in oocytes but expressed in sperm, which delivers it to the oocyte during fertilization [41, 42]. The sperm-born miR-34c has been proposed to regulate maternal mRNA degradation and preimplantation embryonic development in mice [42]. Studies on murine testes and zebrafish oocytes showed that silencing of miR-34c affects the expression of gene targets, including apoptotic gene B-cell lymphoma 2 (*Bcl-2*) [43, 44]. A similar phenotype has been shown in murine zygotes, where effective inhibition of miR-34c enhanced *Bcl-2* expression, suppressing the first zygotic division [41]. The same authors observed an antiproliferative function of *Bcl-2* mediated by p27 (negative cell-cycle regulator protein) when overexpressed in those zygotes. Considering this, it might be justifiable to assume that the role of miR-34c during embryo development is to promote cell proliferation. Consistently, our functional analysis using miR-34c mimics during IVM resulted in higher TCN, ICM, and ICM/TCM ratio than the control but did not affect cleavage or blastocyst yield. A similar outcome was obtained when miR-34c siRNA (small interfering RNA) was injected into human zygotes, resulting in a significantly lower rate of high-quality embryos with slower development kinetics than the control group [39]. Notably, inhibition of embryonic cleavage was not observed after the treatment with siRNA [39]. Moreover, as miRNA mimics remain stable during in vitro embryo culture procedure [29] and penetrate into the oocyte (as we have shown), it is likely that miR-34c regulates gene expression at an earlier developmental stage, before embryonic genome activation (EGA) takes place. In bovine embryos, EGA occurs at the 8-cell stage and is connected to maternal mRNA degradation [55]. The miRNA expression profile of matured oocytes is similar to the one in embryos before EGA but differs in embryos after EGA [56]. Mammalian oocytes harbor miRNAs, yet these molecules do not actively repress their mRNA targets or induce mRNA degradation [57]. This functional absence might stem from inadequate miRNA concentrations to effectively target mRNAs and maternal mRNAs' abundance within fully developed oocytes [58]. We speculate that supplementing miR-34c mimic to IVM medium increased its concentration within the oocytes, potentially affecting the transcriptome around EGA, which resulted in high-quality embryos. In cattle, miR-34c is also expressed by the oocyte at the germinal vesicle and metaphase II stage and in 2-cell embryos [40], where it improves the developmental competence of embryos generated by somatic cell nuclear transfer [45]. Interestingly, our analysis revealed that bta-miR-34c increases its expression as follicles grow, with a higher bta-miR-34c expression observed in LEY compared to SEY and in LEN compared to SEN. With respect to follicle growth, the identification of miRNA profiles from follicular fluid-derived EVs has been previously described in cattle [59]. This study, consistent with our observations, reported higher bta-miR-34c expression from follicular fluid-derived EVs as antral follicles grow (from 6–9 mm follicles to > 9 mm follicles) [59].

Besides bta-miR-34c, several other candidate miRNAs were identified, such as bta-miR-141 and bta-miR-200a/b, belonging to the miR-8 family, detected across both LEY vs. LEN and LSEY vs. LSEN comparisons. The microRNAs from this family are conserved across vertebrates and have a similar 5’ end sequence [60]. These miRNAs have been proposed to regulate oocyte maturation, ovulation, cell differentiation, and division, including a role in epithelial-mesenchymal transition during the first stages of embryo development [61, 62]. Moreover, low expression of miR-141 and miR-200c in granulosa cells of polycystic ovarian syndrome patients was related to increased pregnancy complications [63]. Our findings support the established role of the miR-8 family in influencing oocyte developmental competence, as reported previously [60–63]. Different miRNA profiles in EVs from competent follicular fluid compared to non-competent follicular fluid in small follicles (SEN vs. SEY) were also detected. Interestingly, miR-29c (upregulated in SEY) regulates porcine granulosa cell apoptosis and female fertility [64]. MicroRNA-29c was also upregulated in LEN when compared to SEN. These results suggest that miR-29c is a key miRNA in determining oocyte and follicular somatic cell fate. Overall, few novel DE miRNAs with matched bovine seed regions or homologs with other species were identified. This observation indicates that most of the identified novel DE miRNAs found are unreported in *Bos taurus*. It is important to acknowledge certain limitations of our study. While miR-34c mimic enhanced oocyte competence when matured in vitro, the precise mechanism by which this miRNA could regulate oocyte quality is still unclear. Moreover, our study focused on bovine oocytes, and extrapolating these findings to other species may require caution due to potential species-specific differences in reproductive biology.

## Conclusion

The findings presented herein indicate that EVs isolated from ovarian follicular fluid contain different miRNA cargo associated with the corresponding oocyte’s developmental fate. These miRNAs will facilitate the development of non-invasive biomarkers to predict oocyte developmental competence, which could enhance ART outcomes. Additionally, our results have established that bta-miR-34c present in the follicular fluid environment during oocyte growth might contribute to enhancing embryo quality. Besides advancing our understanding of oocyte biology, our results suggest promising directions for further research, including the potential integration of miRNAs into ART protocols to improve clinical outcomes.

### Supplementary Information

**Additional file 1**: **Table S1**. Cumulus-oocyte expansion assessment and embryo development of oocytes after individual follicle aspiration (FA) and follicle dissection (FD). **Table S2**. Genomic location of differentially expressed miRNAs identified in EVs derived from follicular fluid of large follicles containing competent or non-competent oocytes (LEY vs. LEN). **Table S3**. Genomic location of differentially expressed miRNAs identified in EVs derived from follicular fluid of small follicles containing competent or non-competent oocytes (SEY vs. SEN). **Table S4**. Genomic location of differentially expressed miRNAs identified in EVs from follicular fluid of small and large follicles containing competent and non-competent oocytes (LSEY vs. LSEN). **Table S5**. Genomic location of differentially expressed miRNAs identified in EVs derived from follicular fluid of large and small follicles containing competent oocytes (SEY vs. LEY). **Table S6**. Genomic location of differentially expressed miRNAs identified in EVs derived from follicular fluid of large and small follicles containing non-competent oocytes (LEN vs. SEN).

**Additional file 2**: **Fig. S1**. Description of data: GO enrichment of DE miRNAs derived from EVs in LEY vs. LEN. Utilizing Schlicker’s Relevance method, we computed semantic similarity scores among notable GO terms within their distinct categories—biological process (BP), cellular component (CC), and molecular function (MF). These resultant GO terms underwent clustering through binary cut enrichment and subsequent categorization labeled as (**a**) BP, (**b**) MF, and (**c**) CC.

**Additional file 3**: **Fig. S2**. GO enrichment of DE miRNAs derived from EVs in SEY vs. SEN. Utilizing Schlicker’s Relevance method, we computed semantic similarity scores among notable GO terms within their distinct categories—biological process (BP), cellular component (CC), and molecular function (MF). These resultant GO terms underwent clustering through binary cut enrichment and subsequent categorization labeled as (a) BP, (b) MF, and (c) CC.

**Additional file 4**: **Fig. S3**. Description of data: GO enrichment of DE miRNAs derived from EVs in SEY vs. LEY. Utilizing Schlicker’s Relevance method, we computed semantic similarity scores among notable GO terms within their distinct categories—biological process (BP), cellular component (CC), and molecular function (MF). These resultant GO terms underwent clustering through binary cut enrichment and subsequent categorization labeled as (a) BP, (b) MF, and (c) CC.

**Additional file 5**: **Fig. S4**. GO enrichment of DE miRNAs derived from EVs in SEN vs. LEN. Utilizing Schlicker’s Relevance method, we computed semantic similarity scores among notable GO terms within their distinct categories—biological process (BP), cellular component (CC), and molecular function (MF). These resultant GO terms underwent clustering through binary cut enrichment and subsequent categorization labeled as (a) BP, (b) MF, and (c) CC.

**Additional file 6**: **Fig. S5**. Visualization of microRNA mimics accumulation in the ooplasm of denuded oocyte after in vitro maturation of cumulus-oocyte complexes (COCs). Bright-field and fluorescence images of oocyte after removal of cumulus cells, obtained from COCs matured in vitro with or without labeled miRNA mimic supplementation. After 22 h of in vitro maturation, along with labeled mimics (5′ FAM, green) or PBS (Control), cumulus cells were removed by pipetting, and the denuded oocytes were fixed and stained with Hoechst (blue) to visualize the nuclei. The merged image demonstrates the uptake of green fluorescent–labeled mimics by the oocyte. A control with PBS incubated with bovine COCs showed no green fluorescence in the corresponding denuded oocyte.

## Data Availability

The miRNA sequencing datasets generated during the current study were deposited in the National Center for Biotechnology Information (NCBI) Gene Expression Omnibus (GEO) database with accession number GSE250281.

## Abbreviations

AC: Apoptotic cells
ART: Artificial reproductive technologies
COC: Cumulus-oocyte complex
DE: Differentially expressed
EGA: Embryonic genome activation
EV: Extracellular vesicle
FA: Follicle aspiration
FD: Follicle dissection
ICM: Inner cell mass
IVM: In vitro maturation
L: Large
LEY: Large follicle embryo yes
LEN: Large follicle embryo no
LSEY: Large and small embryo yes
LSEN: Large and small embryo no
miRNA: MicroRNA
NC: Negative control
PBS: Phosphate-buffered saline
S: Small
SEY: Small follicle embryo yes
SEN: Small follicle embryo no
TCM: Tissue culture medium
TCN: Total cell number
TE: Trophectoderm cells
