# Supplementary figures and images for: From follicle to blastocyst: microRNA-34c from follicular fluid-derived extracellular vesicles modulates blastocyst quality
Source: J Anim Sci Biotechnol. 2024 Aug 4;15:104. doi: 10.1186/s40104-024-01059-8 (PMC11298084; doi:10.1186/s40104-024-01059-8)

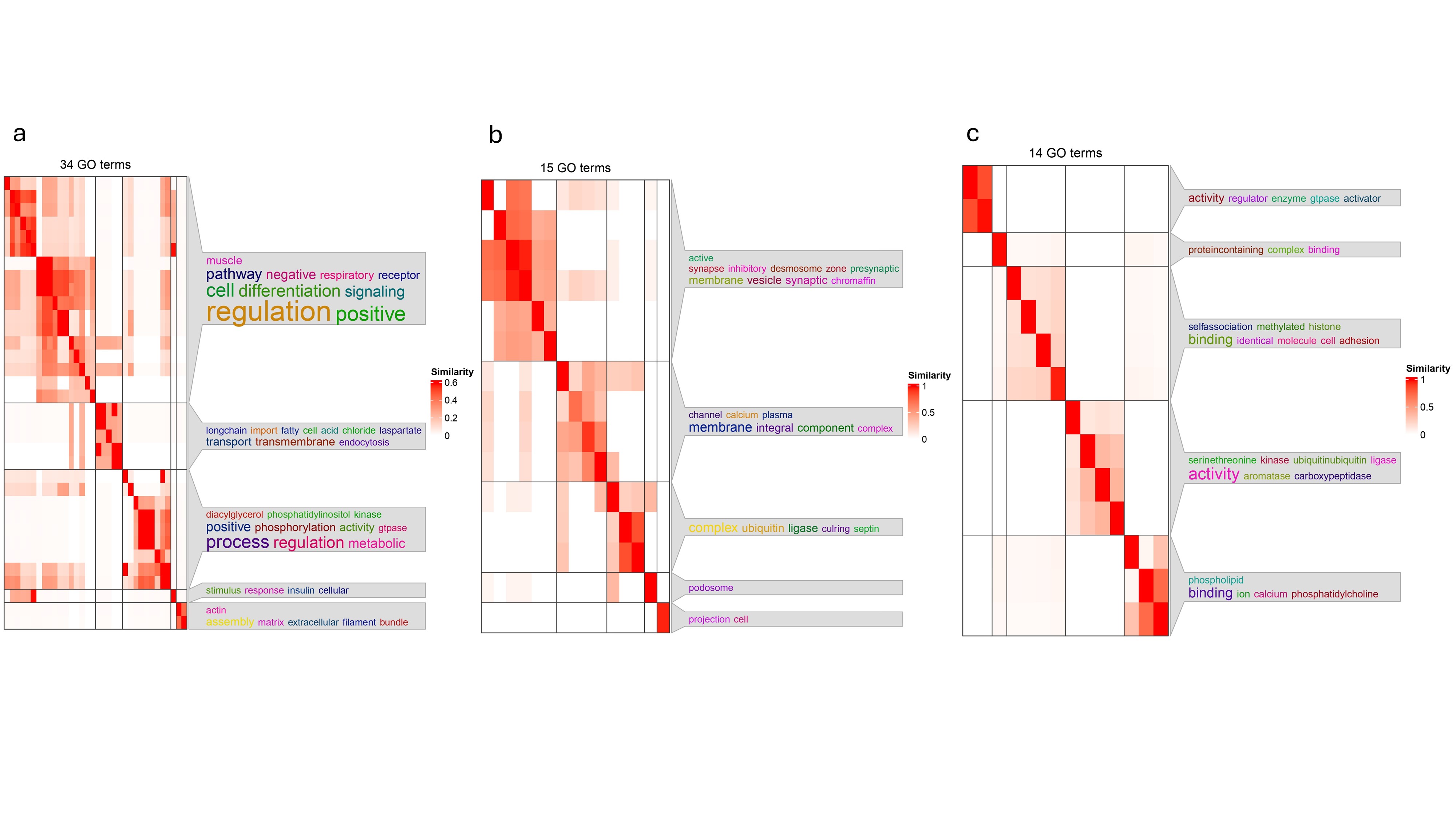

Supplement: Supplementary file 2 — Additional file 2: Fig. S1. Description of data: GO enrichment of DE miRNAs derived from EVs in LEY vs. LEN. Utilizing Schlicker’s Relevance method, we computed semantic similarity scores among notable GO terms within their distinct categories—biological process (BP), cellular component (CC), and molecular function (MF). These resultant GO terms underwent clustering through binary cut enrichment and subsequent categorization labeled as (a) BP, (b) MF, and (c) CC. [file 40104_2024_1059_MOESM2_ESM.jpg]

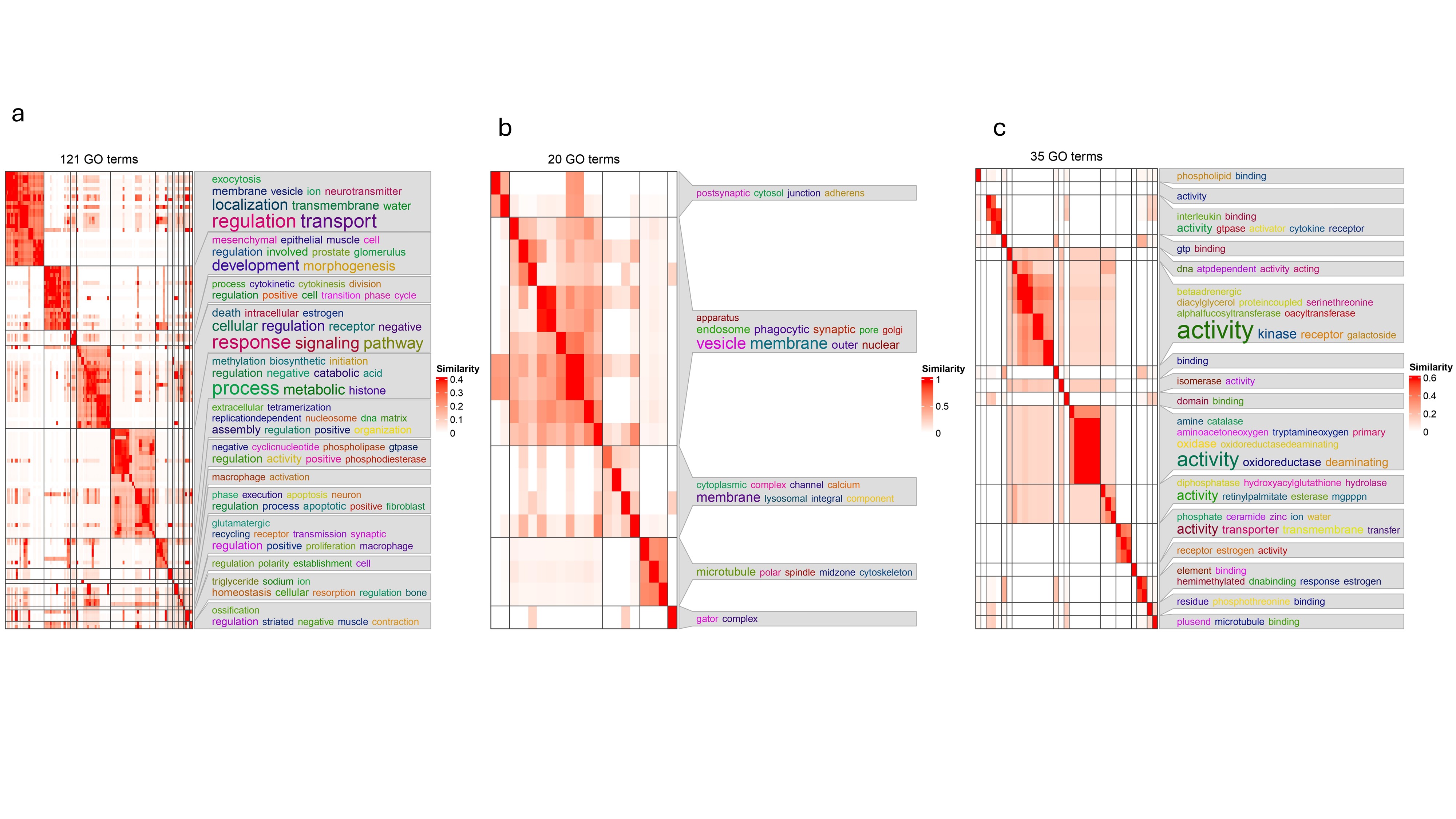

Supplement: Supplementary file 3 — Additional file 3: Fig. S2. GO enrichment of DE miRNAs derived from EVs in SEY vs. SEN. Utilizing Schlicker’s Relevance method, we computed semantic similarity scores among notable GO terms within their distinct categories—biological process (BP), cellular component (CC), and molecular function (MF). These resultant GO terms underwent clustering through binary cut enrichment and subsequent categorization labeled as (a) BP, (b) MF, and (c) CC. [file 40104_2024_1059_MOESM3_ESM.jpg]

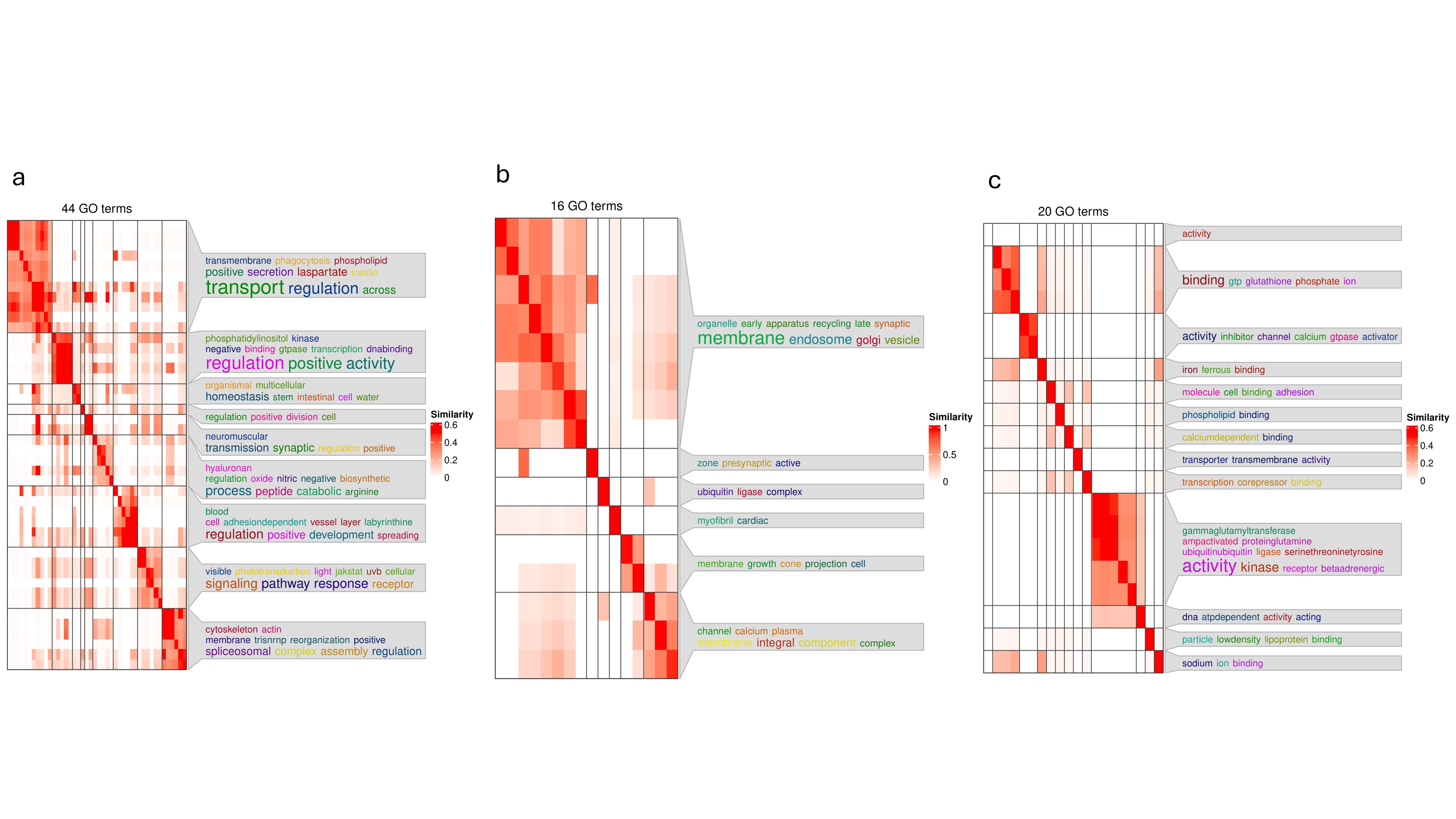

Supplement: Supplementary file 4 — Additional file 4: Fig. S3. Description of data: GO enrichment of DE miRNAs derived from EVs in SEY vs. LEY. Utilizing Schlicker’s Relevance method, we computed semantic similarity scores among notable GO terms within their distinct categories—biological process (BP), cellular component (CC), and molecular function (MF). These resultant GO terms underwent clustering through binary cut enrichment and subsequent categorization labeled as (a) BP, (b) MF, and (c) CC. [file 40104_2024_1059_MOESM4_ESM.jpg]

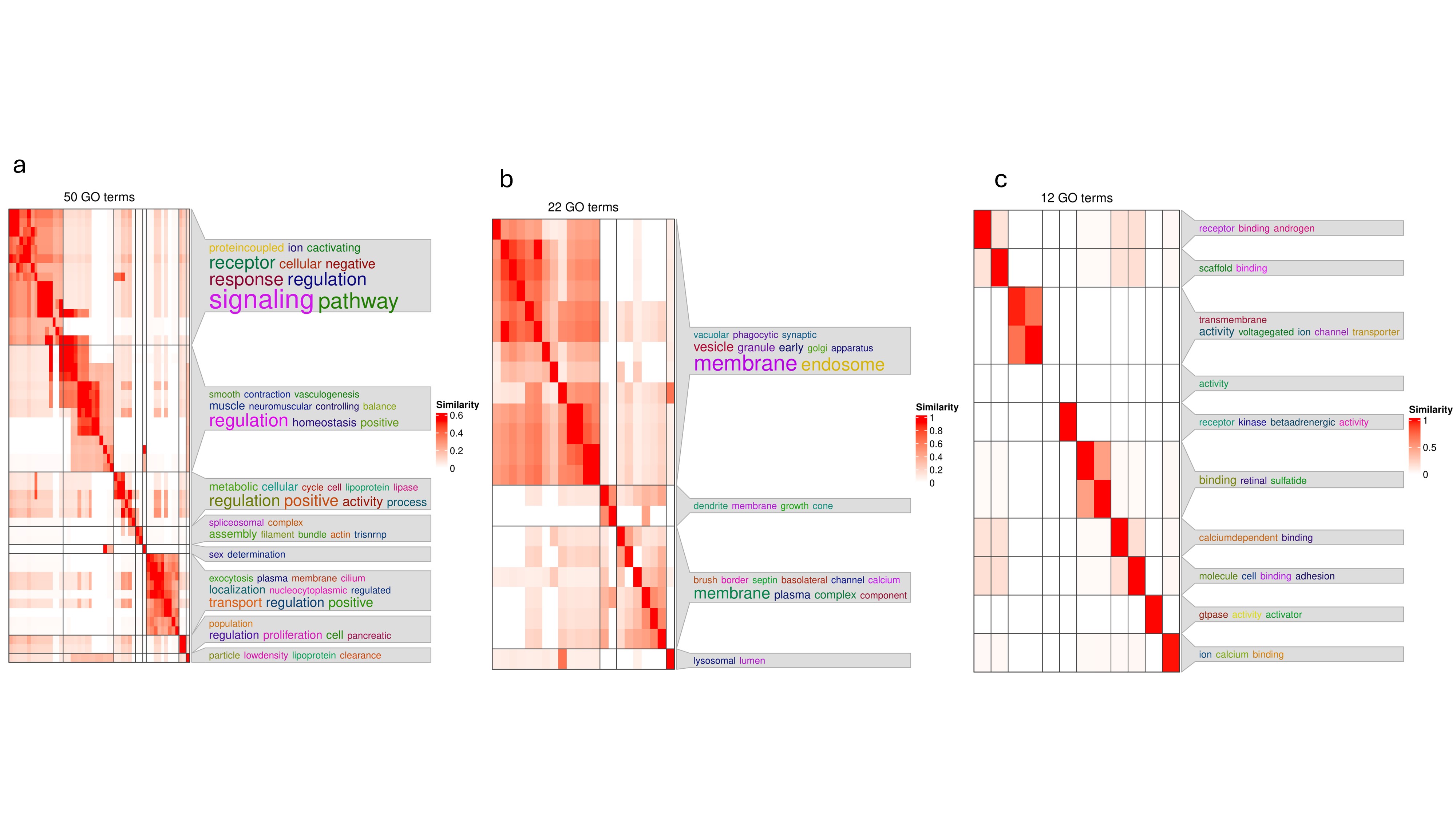

Supplement: Supplementary file 5 — Additional file 5: Fig. S4. GO enrichment of DE miRNAs derived from EVs in SEN vs. LEN. Utilizing Schlicker’s Relevance method, we computed semantic similarity scores among notable GO terms within their distinct categories—biological process (BP), cellular component (CC), and molecular function (MF). These resultant GO terms underwent clustering through binary cut enrichment and subsequent categorization labeled as (a) BP, (b) MF, and (c) CC. [file 40104_2024_1059_MOESM5_ESM.jpg]

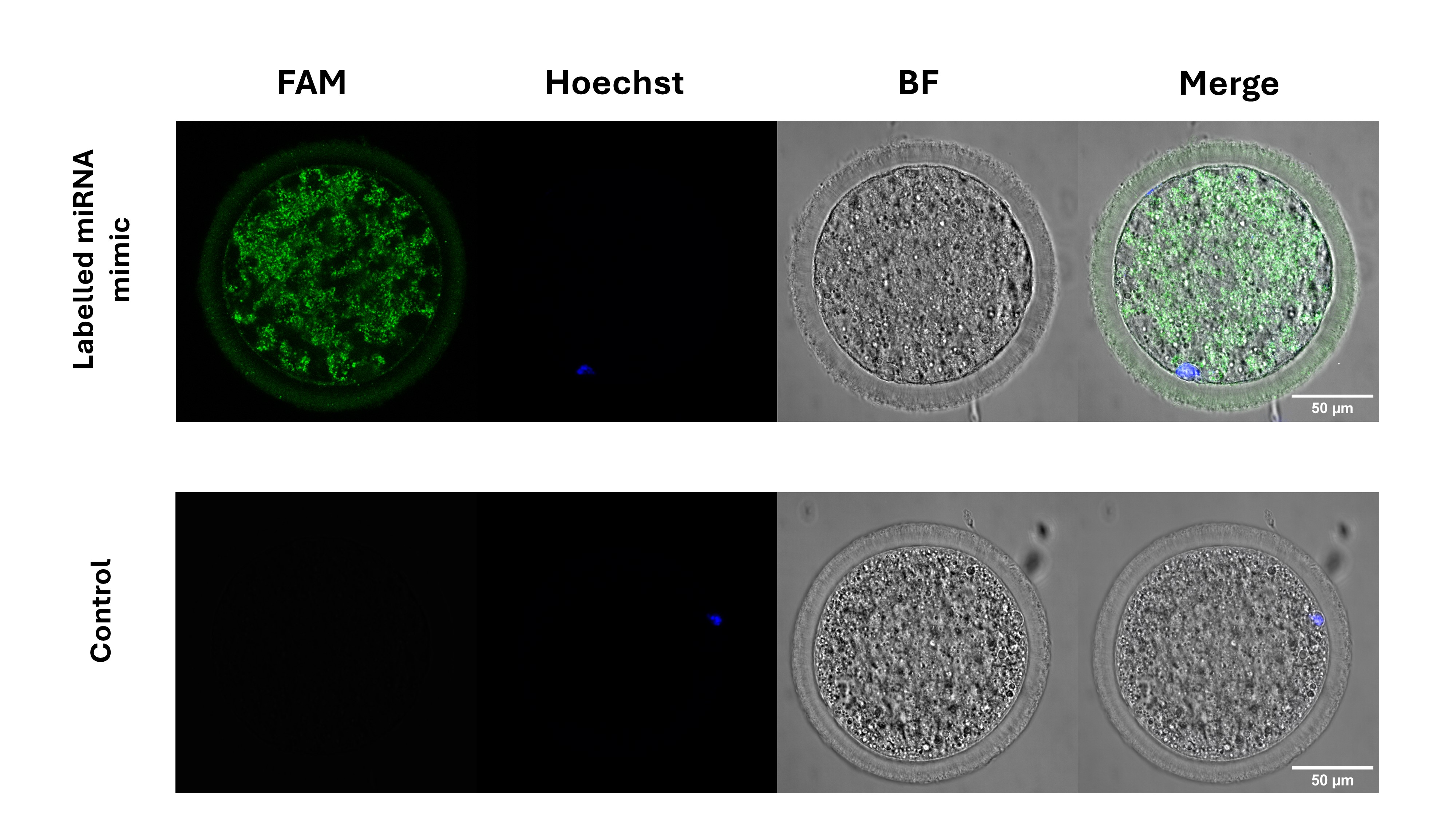

Supplement: Supplementary file 6 — Additional file 6: Fig. S5. Visualization of microRNA mimics accumulation in the ooplasm of denuded oocyte after in vitro maturation of cumulus-oocyte complexes (COCs). Bright-field and fluorescence images of oocyte after removal of cumulus cells, obtained from COCs matured in vitro with or without labeled miRNA mimic supplementation. After 22 h of in vitro maturation, along with labeled mimics (5′ FAM, green) or PBS (Control), cumulus cells were removed by pipetting, and the denuded oocytes were fixed and stained with Hoechst (blue) to visualize the nuclei. The merged image demonstrates the uptake of green fluorescent–labeled mimics by the oocyte. A control with PBS incubated with bovine COCs showed no green fluorescence in the corresponding denuded oocyte. [file 40104_2024_1059_MOESM6_ESM.jpg]
